# Plastic Surgery Tourism: Complications, Costs, and Unnecessary Spending?

**DOI:** 10.1093/asjof/ojad113

**Published:** 2023-12-21

**Authors:** Danielle Hery, Brandon Schwarte, Krishna Patel, John O Elliott, Susan Vasko

## Abstract

**Background:**

The liability of plastic surgery tourism in patient health and postoperative resource allocation is significant. Procedures completed within the context of medical tourism often lack rigorous quality assurance and provide limited preoperative evaluation or postoperative care. Not only does this jeopardize the patient's well-being, but it also increases the financial burden and redirects invaluable resources domestically through often unnecessary diagnostic tests and hospitalizations.

**Objectives:**

This manuscript will examine the complications and associated costs following plastic surgery tourism and highlight unnecessary expenses for patients with outpatient complications.

**Methods:**

A retrospective review was conducted of all patients 18 years or older who underwent destination surgery and were seen within 1 year postoperatively in consultation with plastic surgery at our health system between January 11, 2015 and January 7, 2022. Patient admissions were reviewed and deemed necessary or unnecessary after review by 2 physicians.

**Results:**

The inclusion criteria were met by 41 patients, of whom hospitalization was deemed necessary in 28 patients vs unnecessary in 13 patients. The most common procedures included abdominoplasty, liposuction, breast augmentation, and “Brazilian butt lift.” The most common complications were seroma and infection. Patients deemed to have a necessary admission often required at least 1 operation, were more likely to need intravenous antibiotics, were less likely to have the diagnosis of “pain,” necessitated a longer hospitalization, and incurred a higher cost. The total financial burden was $523,272 for all 41 patients.

**Conclusions:**

Plastic surgery tourism poses substantial health risks, the morbidities are expensive, and it strains hospital resources.

**Level of Evidence: 5:**

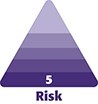

Medical tourism is defined as when a patient travels, domestically or internationally, specifically to receive medical care. It has witnessed a surge in popularity over the last few decades, especially in plastic surgery.^1-3^ Unfortunately, plastic surgery tourism can have significant ramifications. Multiple studies have demonstrated increased mortality with plastic surgery tourism due to the lack of appropriate postoperative care, with complications such as uncontrolled pain, infection, wound dehiscence, or poor aesthetic outcome that the patient must deal with independently at home.^3-7^ Often, these complications require admission into the hospital, surgical intervention, and long-term follow-up and can pose a huge financial burden, with costs ranging from $26,000 to $154,000.^3^ Patients travel for many reasons, including increased privacy, lower wait times, interest in combining a vacation with surgery, and most commonly, decreased cost.^4,8,9^ Unfortunately, quality and safety measures are often compromised to allow for a lower cost of surgery, often predisposing the patient to a much more expensive complication.^7^ More importantly, an individual's health and life are jeopardized, deeming medical tourism unsafe by many, with efforts in place to improve safety guidelines and public awareness.^1,7^

An Australian study showing significant financial burden and multiple endangered lives questions not the surgical skill or technique, but often the lack of perioperative care, thus indicating that the delivery model of medical tourism is inadequate.^10^ Despite the ethical dilemma of treating patients without being involved in their surgery, prioritizing patient safety and delivering prompt, dependable care aligns with the American Board of Plastic Surgeons Code of Ethics and enhances the well-being of society at large.^11,12^ Patients with complications from plastic surgery tourism should be seen and evaluated by a plastic surgery team.

Patients of plastic surgery tourism with complications are often seen, diagnosed, and admitted into the hospital without ever seeing their operating surgeon. One study showed that only 26% of patients received a postoperative appointment.^11^ Without a “home” surgeon, patients present to the emergency department (ED) for any complication or question. Minor morbidities, such as fat necrosis, seroma, superficial dehiscence, and pain, are evaluated in the emergency room (ER) without the guidance of a plastic surgeon and can undergo extensive unnecessary diagnostic and therapeutic interventions for issues that can be resolved with an office visit or phone call. Only later are they seen by a plastic surgeon and generally discharged, resulting in unnecessary medical expenses. The extent of useless tests and studies has been well documented and with ER visits on the rise, some insurance companies have gone to the extreme of denying coverage for inappropriate/nonemergent visits.^13-15^

The purpose of this study is to evaluate the cost of complications from destination surgery and to estimate the cost of unnecessary workup and admissions once these patients return home. Understanding this may not only improve appropriate disposition at the ED through encouraging earlier plastic surgery involvement or use of telemedicine but can also increase public awareness regarding the safety issues associated with medical tourism.^16,17^

## METHODS

A retrospective cohort study was conducted involving all patients ≥18 years who presented to our institution from January 1, 2015 to Janurary 11, 2022 and were seen in plastic surgery consultation within 1 year after undergoing destination surgery. Patients who presented greater than 1 year postoperatively were excluded from the study.

### Patient Encounter Review Process

Dr Vasko is a board-certified plastic surgeon and Dr Hery is a general surgery chief resident. Each patient case and admission were reviewed by both of these authors and determined to be necessary or unnecessary based on whether an inpatient medical or procedural intervention was necessitated. An unnecessary admission was defined as an inpatient admission or ER visit where treatments and procedures were performed that would normally be performed in an outpatient setting. For example, if the patient is started on oral antibiotics (something that could be done in an office visit) and discharged that would be considered unnecessary. On the other hand, if a patient was admitted and plastic surgery recommended further evaluation or surgery, this was deemed as a necessary admission. Since some patients could have a combination of both inpatient and outpatient interventions done simultaneously (eg, starting oral antibiotics and need for surgery), the assignment of whether this was a necessary or unnecessary admission was based on the admission as a whole, such that if a necessary intervention was performed, the admission was deemed necessary. Incongruence between the 2 physician raters about whether an admission was necessary or unnecessary was discussed until a consensus was obtained.

### Statistical Analysis

The study population was summarized using means, standard deviations, percentages, and ranges. Hospitalization data such as whether the patient saw a provider prior to presentation, diagnostic testing, interventions performed by the plastic surgery or other teams, if there were follow-up or repeat admissions, and the cost of the hospital admission were collected. The actual cost incurred by our institution was extracted from the administrative record for each patient encounter and calculated based on relative value units, a standardized indicator of the value of services. All analyses were conducted in SPSS Version 25.

## RESULTS

A total of 41 patients met the inclusion criteria. Of these, admission was deemed necessary in 28 (68%) patients and unnecessary in 13 (32%) patients. As seen in Table 1, the majority of the patients (*n* = 40; 98%) were female (1 male, *n* = 1, 2%) and were insured through Medicaid (*n* = 29; 70%). The ages in the necessary admission group ranged from 27 to 56 years old, with the average being 37 years old, and the age range for the unnecessary admission group was 22 to 55 years old, with the average being 39 years old.

**Table 1.** Patient Characteristics

	Unnecessary	Necessary	
Characteristic	(*n* = 13)	(*n* = 28)	*P*-value
Age, mean ± SD	37.2 ± 8.7	38.8 ± 8.6	.586
Sex, % (*n*)			1.000
Female	100 (13)	96.4 (27)	
Male	0 (0)	3.6 (1)	
Insurance payor, % (*n*)			.299
Medicare	7.7 (1)	0 (0)	
Medicaid	69.2 (8)	71.4 (20)	
Self-pay	0 (0)	10.7 (3)	
Private insurance	23.1 (3)	17.9 (5)	
Comorbid conditions, % (*n*)			
Hypertension	15.4 (2)	28.6 (8)	.458
Diabetes	0 (0)	3.6 (1)	1.000
Coronary artery disease	0 (0)	0 (0)	—
Congestive heart failure	0 (0)	3.6 (1)	1.000
Anxiety/depression	23.1 (3)	39.3 (11)	.481
History of smoking, % (*n*)	38.5 (5)	32.1 (9)	.734
Blood thinner use, % (*n*)	7.7 (1)	0 (0)	.317

SD, standard deviation.

Table 2 demonstrates the most common destination procedures, with abdominoplasty (*n* = 31; 76%), liposuction (*n* = 23; 56%), breast augmentation (*n* = 11; 27%), and/or Brazilian butt lift (*n* = 10; 24%). The top 3 most common surgeries in this study are the same as the top surgical procedures completed nationally in the United States in 2021.^18^ Over 60% of patients in both groups had no follow-up with their surgeon after they left their destination (Table 2).

**Table 2.** Original Surgery Characteristics

	Unnecessary	Necessary	
Characteristic	(*n* = 13)	(*n* = 28)	*P*-value
Procedure, % (*n*)			
Breast augmentation	30.8 (4)	25.0 (7)	.719
Abdominoplasty	84.6 (11)	71.4 (20)	.458
Liposuction	69.2 (9)	50.0 (14)	.321
Gluteal lift	30.8 (4)	21.4 (6)	.698
Other	0 (0)	17.9 (5)	.160
Location of surgery, % (*n*)			.409
Arizona	0 (0)	3.6 (1)	
California	7.7 (1)	0 (0)	
Dominican Republic	15.4 (2)	32.1 (9)	
Florida	76.9 (10)	42.9 (12)	
Iowa	0 (0)	3.6 (1)	
Louisiana	0 (0)	3.6 (1)	
Michigan	0 (0)	3.6 (1)	
Ohio	0 (0)	7.1 (2)	
Texas	0 (0)	3.6 (1)	
Had follow-up with original surgeon, % (*n*)			.785
No	61.5 (5)	60.7 (17)	
Yes	38.5 (5)	35.7(10)	
Unknown	0 (0)	3.6 (1)	

The most common complications were seroma (*n* = 23; 56%) and infection (*n* = 21; 51%). Patients deemed to have a necessary admission were more likely to need intravenous antibiotics (42.9% vs 0%, *P* = .007), often required at least 1 operation (64.3% vs 0%, *P* < .001), get blood or body fluid cultures taken while admitted, have a longer hospital stay, and more likely to be seen for follow-up (Tables 3, 4). The average short-interval follow-up time for necessary admission patients was 7 days and for unnecessary admission patients was 11 days. The range for necessary patients was 2 to 161 days and for the unnecessary patients was 4 to 52 days.

**Table 3.** Admission Characteristics

	Unnecessary	Necessary	
Characteristic	(*n* = 13)	(*n* = 28)	*P*-value
Days since original surgery, mean ± SD	30.9 ± 26.1	31.5 ± 24.7	.942
Saw medical provider before coming to the hospital, % (*n*)	7.7 (1)	32.1 (9)	.129
Plastic surgery interventions, % (*n*)			
None	15.4 (2)	3.6 (1)	.232
Oral antibiotics	69.2 (9)	42.9 (12)	.181
IV antibiotics	0 (0)	42.9 (12)	.007
Surgery	0 (0)	64.3 (18)	<.001
Minor procedure	30.8 (4)	25.0 (7)	.719
Hospital workup, % (*n*)			
CT scan	100 (13)	96.4 (27)	1.000
Ultrasound	15.4 (2)	7.1 (2)	.579
Culture	23.1 (3)	71.4 (20)	.008

CT, computer tomography; SD, standard deviation.

**Table 4.** Patient Outcomes

	Unnecessary	Necessary	
Characteristic	(*n* = 13)	(*n* = 28)	*P*-value
Complications from original surgery, % (*n*)			
Skin necrosis	0 (0)	14.3 (4)	.288
Pain	38.5 (5)	3.6 (1)	.008
Infection	30.8 (4)	60.7 (17)	.100
Seroma	38.5 (5)	64.3 (18)	.179
Wound dehiscence	30.8 (4)	28.6 (8)	1.000
Contour abnormalities	0 (0)	3.6 (1)	1.000
Other	7.7 (1)	14.3 (4)	1.000
Mortality	0 (0)	0 (0)	—
Transfusion, % (*n*)	0 (0)	7.1 (2)	1.000
ICU stay, % (*n*)	0 (0)	0 (0)	—
Seen for follow-up, % (*n*)	38.5 (5)	75.0 (21)	.038
Repeat hospitalization, % (*n*)	15.4 (2)	28.6 (8)	.458
Length of stay, mean ± SD	1.8 ± 1.4	5.3 ± 3.9	.003
Cost of admission, mean ± SD	$3828 ± $2540	$16,911 ± $14,018	.001

ICU, intensive care unit; SD, standard deviation.

Patients deemed necessary were less likely to have the diagnosis of “pain” (3.6% vs 38.5%, *P* = .008) and had a longer average length of stay (5.3 vs 1.8 days, *P* = .003). The average cost for a necessary admission was significantly higher ($16,911 vs $3828, *P* = .001; Table 4). Total cost was $523,272 for all 41 patients.

## DISCUSSION

Medical tourism is defined as the act of traveling either domestically or internationally with the intent to obtain healthcare. Medical tourism has witnessed an enormous surge in popularity and though difficult to verify, conservative estimates of the global market value in 2022 range from $10 to $36 billion, with a compound annual growth rate of 14% to 31%.^19,20^ Cosmetic surgeries are among the most common destination surgeries performed and typically consist of abdominoplasty, breast augmentation, gluteal augmentation, and liposuction.^21-23^ Popular countries for destination surgery for patients residing in the United States include the Dominican Republic, Mexico, Columbia, Turkey, and Thailand likely as a result of lower costs, decreased wait times, increased privacy, and prospects for a combined vacation.^5,22-24^ Fifty-three percent of the patients in our study had their procedures performed in Florida, a state which has been known for its lenient policies and regulations regarding outpatient plastic surgery centers and the physicians who are performing them. Florida law allows any physician to perform any procedure as long as they have the patient's permission, regardless of the physicians training or prior experience. An increase in publicity about several out-of-state medical tourists’ complications and even mortalities, particularly following BBL's (Brazilian butt lift) or gluteal lift and body contouring procedures, prompted the state of Florida to impose stricter regulations. As of July 2023, the state's first plastic surgery bill was passed mandating: the use of ultrasound guidance during gluteal fat grafting procedures as well as video documentation of ultrasound-guided technique in the patient's chart, fat grafting may only be performed by the operating surgeon and may not be delegated, in-person examinations by the operating surgeon prior to the day of surgery, office inspections by the Department of Health must be passed prior to opening and will undergo annual reinspection. These new laws have been instituted in an attempt to optimize patient safety and hold physicians to a higher standard but how this will ultimately affect the complication rates and continually increasing popularity of surgical tourism has yet to be delineated.^25^

Unfortunately, decreased procedural costs of destination plastic surgery may be due to less stringent quality assurance, as demonstrated by the lack of informed consent reported in 1 study in 50% of patients.^26^ The lack of appropriate preoperative medical evaluation raises concerns for patient selection and limits optimization of controllable factors that can predispose to poor outcomes (eg, smoking cessation, determining expectations). We found that over one-third (34%) of our patients smoked tobacco. All patients are screened in the ED at our institution for nicotine use, but it is unclear as to whether they were asked about smoking prior to their original surgery. Active nicotine use in these patients more than likely contributed to adverse outcomes. Moreover, language and cultural barriers can exacerbate the vulnerability felt by these patients as they are recovering in an unfamiliar environment. Postoperative care is often limited or truncated and nearly half of the patients in 1 study report no postoperative evaluation by their operating surgeon (60% in our study).^26^ Also, patients following destination surgery often return home without adequate recovery and suffer risks that domestic patients are not typically exposed to (eg, long flights).^7^ In our opinion, patients who simply travel either domestically or abroad in pursuant of better medical care (eg, higher expertise) are not subjected to the same vulnerabilities as the aforementioned population, as these patients often undergo likely more stringent perioperative evaluation. Ambiguity is, however, further increased when itinerant surgeons travel to perform destination surgeries and require patients to receive their perioperative care at these locations.

The most common complications following plastic surgery tourism in our study were seroma (56%) and infection (51%), with some patients experiencing both, and are similar to the distributions of adverse outcomes described in the literature.^22,27^ Interestingly, the incidence of “pain” was lower in those who required admission relative to those who were managed in the outpatient setting (3.6% vs 38.5%, *P* = .008), suggesting those who were admitted had more complex complications than poor analgesic control, which could easily be managed without an in-hospital stay.

While lower surgical fees may be enticing at first, patients often overlook the costs of complications if they were to arise.^28^ The lack of appropriate postoperative care by the operating surgeon following surgical tourism mandates evaluation by a medical team previously unknown to the patient, predisposing to unnecessary diagnostic testing and interventions. Nearly $1.3 billion was spent in 2017 by the United States in treating complications arising from medical tourism.^29^ The average cost for evaluation in our study is approximately $6180 per patient and is not dissimilar to expense reports published previously ($6000-$26,000).^7,30,31^ Furthermore, as described in other studies, Medicaid was the primary insurance provider for our patients as well.^7,22^ Seventy percent of our patient population was insured by Medicaid which is funded by federal and state taxpayers. A 2016 paper analyzing medical tourism calculated that approximately 15 million US citizens seek healthcare abroad, with an average cost of complications at over $18,000 each an estimated total cost to the US healthcare system was $1.33 billion.^32^

After a case-by-case evaluation by 2 physicians, we determined that 13 (32%) of 41 patients were hospitalized unnecessarily. Unsurprisingly, there was a significant difference in expense between those who were admitted and those whose complications could be managed on an outpatient basis, highlighting the importance of appropriate triaging and early evaluation by the plastic surgery team to minimize unwarranted costs and resource utilization. Although the sensitivity and specificity of diagnostic tests vary by patient and case, typical laboratory testing such as a complete blood count and basic metabolic panel and diagnostic imaging (eg, computer tomography [CT] or ultrasound) may help uncover an underlying infection and guide appropriate management with early antibiotics and/or intervention, and can help significantly decrease total hospital costs and readmission rates.^33^ The patients needing inpatient care were also more likely get blood or body fluid cultures while in the hospital and be started on IV antibiotics, which is expected given cultures are generally only taken with sepsis or with a drainage procedure and IV antibiotics initiated in more septic patients needing inpatient admission. Pain was more likely the chief complaint in patients who did not require admission. Besides 1 patient who required a pain consult and IV pain medications (necessary admission), the 5 other patients (unnecessary admission) had no other complications besides regular postoperative pain and were able to be discharged with oral pain medications. Patients with necessary admission were unsurprisingly likely to have a longer length of stay, given they are generally undergoing procedures/surgeries and receiving more intensive treatment. Because these patients often have more critical complications, potentially receive a surgery, and have a longer length of stay, we believe that they likely become more familiar with the surgeon and have a more difficult recovery after their admission, making them more likely to follow-up with the surgeon as well.

As previously shown, most postoperative complications in these patients can be managed in the outpatient setting and thus prompt involvement of the plastic surgery team in the ER can assist with appropriate triage in these patients.^5,29^

### Limitations

This study utilizes data collected during the patient's clinical course. Clinical data may be recorded differently among providers and carries the risk of being incomplete. The study sample was derived by convenience sampling, and thus it is prone to selection bias and may not be representative of the general population. As only patients with postdestination surgery complications are captured by this study, calculating the true incidence of adverse events is difficult given the nearly impossible task of ascertaining total patient volume in this demographic, which complicates accurate outcome comparisons between domestic and tourist surgical surgeons. Also, it is possible that small complications following destination surgery were addressed by a provider not part of the plastic surgery team and did not require consultation and meet the inclusion criteria. These patient encounters have the possibility of not being captured by this study. Furthermore, the necessity of patient admission may involve some subjectivity, and review by only 2 physicians may not be truly representative of the generalized practice of plastic surgeons across various levels of training, experience, and comfort.

## CONCLUSIONS

Destination plastic surgery confers significant medical complications, including the need for operative procedures, nonsurgical interventions, and days of intravenous antibiotics. Seromas and infections were the most common complications, and most patients did not have follow-up with their original surgeons. The majority of the admissions were deemed to be necessary and were appropriately triaged. Unsurprisingly, these patients contributed to a significantly increased medical expense compared to those whose admission was deemed unnecessary, although both patient subgroups were costly. Whether patients had complications that necessitated admission or not, plastic surgery tourism poses substantial health risks, their morbidities are expensive, and it strains hospital resources.

## References

[1] Melendez MM, Alizadeh K. Complications from international surgery tourism. Aesthet Surg J. 2011;31(6):694–697. doi: 10.1177/1090820X11415977 21813883

[2] Franzblau LE, Chung KC. Impact of medical tourism on cosmetic surgery in the United States. Plast Reconstr Surg Glob Open. 2013;1(7):e63. doi: 10.1097/GOX.0000000000000003 25289258 PMC4174065

[3] International Society of Aesthetic Plastic Surgery. ISAPS . Global Survey 2021: Full Report and Press Releases; 2021. https://www.isaps.org/media/vdpdanke/isaps-global-survey_2021.pdf.

[4] Padilla P, Ly P, Dillard R, Boukovalas S, Zapata-Sirvent R, Phillips LG. Medical tourism and postoperative infections: a systematic literature review of causative organisms and empiric treatment. Plast Reconstr Surg. 2018;142(6):1644–1651. doi: 10.1097/PRS.0000000000005014 30489537

[5] Qureshi AA, Gould DJ, Stevens WG, Fernau J. Report on current experience of ASAPS membership and management of cosmetic tourism complications. Aesthet Surg J Open Forum. 2019;1(2):ojz009. doi: 10.1093/asjof/ojz009 33791605 PMC7671244

[6] Pereira RT, Malone CM, Flaherty GT. Aesthetic journeys: a review of cosmetic surgery tourism. J Travel Med. 2018;25(1):1–8. doi: 10.1093/jtm/tay042[AQ7] 29924349

[7] Venditto C, Gallagher M, Hettinger P, et al Complications of cosmetic surgery tourism: case series and cost analysis. Aesthet Surg J. 2021;41(5):627–634. doi: 10.1093/asj/sjaa092 32291444

[8] Chen LH, Wilson ME. The globalization of healthcare: implications of medical tourism for the infectious disease clinician. Clin Infect Dis. 2013;57(12):1752–1759. doi: 10.1093/cid/cit540 23943826 PMC7107947

[9] Asher CM, Fleet M, Jivraj B, Bystrzonowski N. Cosmetic tourism: a costly filler within the national health service budget or a missed financial opportunity? A local cost analysis and examination of the literature. Aesthetic Plast Surg. 2020;44(2):586–594. doi: 10.1007/s00266-019-01571-7 31832735

[10] Livingston R, Berlund P, Eccles-Smith J, Sawhney R. The real cost of “cosmetic tourism” cost analysis study of “cosmetic tourism” complications presenting to a public hospital. Eplasty. 2015;15:e34. PMID: 26240672 26240672 PMC4522144

[11] Iorio ML, Verma K, Ashktorab S, Davison SP. Medical tourism in plastic surgery: ethical guidelines and practice standards for perioperative care. Aesthetic Plast Surg. 2014;38(3):602–607. doi: 10.1007/s00266-014-0322-6 24797678

[12] American Board of Plastic Surgery . Code of ethics of the American Board of Plastic Surgery; 2018. https://www.abplasticsurgery.org/media/2654/abps-code-of-ethics-final-approved-5-18-18.pdf.

[13] Chou SC, Gondi S, Baker O, Venkatesh AK, Schuur JD. Analysis of a commercial insurance policy to deny coverage for emergency department visits with nonemergent diagnoses. JAMA Netw Open. 2018;1(6):e183731. doi: 10.1001/jamanetworkopen.2018.3731 30646254 PMC6324426

[14] Mafi JN, Russell K, Bortz BA, Dachary M, Hazel WA, Low-Cost FA. High-volume health services contribute the most to unnecessary health spending. Health Aff (Millwood). 2017;36(10):1701–1704. doi: 10.1377/hlthaff.2017.0385 28971913 PMC6727655

[15] Lyu H, Xu T, Brotman D, et al Overtreatment in the United States. PLoS One. 2017;12(9):e0181970. doi: 10.1371/journal.pone.0181970 28877170 PMC5587107

[16] Paik AM, Granick MS, Scott S. Plastic surgery telehealth consultation expedites Emergency Department treatment. J Telemed Telecare. 2017;23(2):321–327. doi: 10.1177/1357633X16639459 27056907

[17] Vyas KS, Hambrick HR, Shakir A, et al A systematic review of the use of telemedicine in plastic and reconstructive surgery and dermatology. Ann Plast Surg. 2017;78(6):736–768. doi: 10.1097/SAP.0000000000001044 28328635

[18] Aesthetic Plastic Surgery National Databank Statistics 2020-2021 . *Aesthet Surg J*. 2022;42(Supplement_1):1–18. doi: 10.1093/asj/sjac116. 35730469

[19] Grand View Research . Medical tourism market size share & trends analysis report by treatment type cosmetic treatment bariatric treatment by service provider private public by country and segment forecasts 2023-2030. Accessed November 18, 2023. https://www.grandviewresearch.com/industry-analysis/medical-tourism-market.

[20] The Business Research Company . Global Medical Tourism Market Report 2022. Accessed November 18, 2023. https://www.thebusinessresearchcompany.com/report/medical-tourisms-global-market-report.

[21] Hummel CE, Klein HJ, Giovanoli P, Lindenblatt N. Complications arising from aesthetic surgery procedures in foreign countries and Switzerland. Swiss Med Wkly. 2023;153(4):40077. doi: 10.57187/smw.2023.40077 37186084

[22] McAuliffe PB, Muss TEL, Desai AA, Talwar AA, Broach RB, Fischer JP. Complications of aesthetic surgical tourism treated in the USA: a systematic review. Aesthetic Plast Surg. 2023;47(1):455–464. doi: 10.1007/s00266-022-03041-z 36315261 PMC9619012

[23] Farid M, Nikkhah D, Little M, Edwards D, Needham W, Shibu M. Complications of cosmetic surgery abroad—cost analysis and patient perception. Plast Reconstr Surg Glob Open. 2019;7(6):e2281. doi: 10.1097/GOX.0000000000002281 31624684 PMC6635218

[24] International Society of Aesthetic Plastic Surgery . ISAPS. International Survey on Aesthetic/Cosmetic Procedures; 2022. https://www.isaps.org/media/a0qfm4h3/isaps-global-survey_2022.pdf.

[25] Health & Human Services Committee and Healthcare Regulation Subcommittee and Busatta Cabrera (CO-SPONSORS) Basabe; López J, Mooney. Health Care Provider Accountability. July 1, 2023. HB 1471. Accessed November 17, 2023. http://laws.flrules.org/2023/307

[26] Robertson EM, Moorman SWJ, Korus LJ. Why do Canadians travel abroad for cosmetic surgery? A qualitative analysis on motivations for cosmetic surgery tourism. Plast Surg (Oakv). 2022;30(4):353–359. doi: 10.1177/22925503211019607 36212104 PMC9537712

[27] McCrossan S, Martin S, Hill C. Medical tourism in aesthetic breast surgery: a systematic review. Aesthetic Plast Surg. 2021;45(4):1895–1909. doi: 10.1007/s00266-021-02251-1 33876284 PMC8054849

[28] McMahon ME, Gressmann K, Martin-Smith JD. An objective analysis of quality and readability of online information for patients seeking cosmetic surgery abroad. J Plast Reconstr Aesthet Surg. 2023;81:88–90. doi: 10.1016/j.bjps.2023.04.051 37121048

[29] Adabi K, Stern CS, Weichman KE, et al Population health implications of medical tourism. Plast Reconstr Surg. 2017;140(1):66–74. doi: 10.1097/PRS.0000000000003459 28654593

[30] Rafeh S, Tara M C, Michael F, et al An analysis of the cost and impact of cosmetic tourism and its associated complications: a multi institutional study. Surgeon. 2022;20(6):339–344. doi: 10.1016/j.surge.2021.12.007 35012867

[31] Henry N, Abed H, Warner R. The ever-present costs of cosmetic surgery tourism: a 5-year observational study. Aesthetic Plast Surg. 2021;45(4):1912–1919. doi: 10.1007/s00266-021-02183-w 33625528

[32] Adabi K, Stern C, Colasante C, et al Abstract: medical tourism and its impact on plastic surgery in the United States. Plast Reconstr Surg Glob Open. 2016;4(Supplement_9):28–29. doi: 10.1097/01.GOX.0000511261.68101.f2

[33] Adabi K, Stern CS, Kinkhabwala CM, et al Early surgical management of medical tourism complications improves patient outcomes. Plast Reconstr Surg. 2020;145(5):1147–1154. doi: 10.1097/PRS.0000000000006728 32332529

